# Supplementary material for: A Stackelberg-based repurchase strategy for rail freight options (BRFO)
Source: PLoS One. 2024 Sep 20;19(9):e0307215. doi: 10.1371/journal.pone.0307215 (PMC11414986; doi:10.1371/journal.pone.0307215)
Supplement: Attachment — Submitted filename: Recommendation.pdf [file pone.0307215.s002.pdf]

General Comments

The manuscript presents a valuable study on a Stackelberg-based buyback approach towards rail freight options. While the work is commendable, there are several areas that require minor revisions to enhance the overall quality and clarity of the paper. The main issues include a brief conclusion and discussion section, insufficient hypotheses regarding future model improvements, and inadequate explanations of the assumptions regarding the distributions of variables in the equations. Below are my detailed comments and suggestions for revisions.

Detail Comments

Conclusion and Discussion:

The conclusion section is relatively brief and lacks depth. It is essential to provide a more comprehensive discussion of the results and their implications. This should include a clear summary of the findings, their relevance, and any limitations encountered.
Additionally, the potential future directions for improving the model need to be more thoroughly hypothesized. Discussing these aspects will provide readers with a clearer understanding of the study's significance and its possible extensions.

Assumptions in Equations:

The manuscript introduces several variables in the equations and assumes that they follow specific distributions. However, the explanations for these assumptions are not sufficiently detailed.
Please provide a more comprehensive rationale for the choice of distributions for these variables. This should include theoretical justification and any empirical evidence supporting these assumptions.

References:

The manuscript would benefit from a more extensive review of related literature. Ensure that all

relevant studies are cited, particularly those that have directly influenced the assumptions and methodologies used in this work.

Conclusion

In conclusion, while the manuscript offers valuable insights and makes a meaningful contribution to the field of rail freight options using a Stackelberg-based buyback approach, several minor revisions are necessary. By expanding the discussion section, providing a more robust hypothesis on future model improvements, and offering detailed explanations for the assumptions in the equations, the manuscript will be significantly strengthened. I encourage the authors to make these revisions to enhance the quality and impact of their work.
