# Supplementary material for: A Stackelberg-based repurchase strategy for rail freight options (BRFO)
Source: PLoS One. 2024 Sep 20;19(9):e0307215. doi: 10.1371/journal.pone.0307215 (PMC11414986; doi:10.1371/journal.pone.0307215)
Supplement: Attachment — Submitted filename: Response to Reviewers.docx [file pone.0307215.s003.docx]

Reviewer #1: A repurchase strategy for the rail freight option based on the Stackelberg game is proposed in this paper. It is necessary to present the work as a research paper, with the following considerations in mind:

1.) The work's title lacks specificity and is presented in a generic format. The title ought to be revised in a concise yet impactful manner to convey the primary subject matter.

Response:Thank you for your suggestion regarding the title. I will revise it to be more specific and impactful, highlighting the concept and application of "BRFO" (Bilateral Rail Freight Option) in a concise manner.

2.) The abstract should encompass a concise explication of the study's motivation and significance.

Response:I will revise the abstract to better explain the motivation and significance of the study, providing a clearer context and background for the research.

3.) In addition to competitive games, the introduction and literature review must cover other game types that are utilised in this domain, including Nash games, differential games, fuzzy games, and others. An analysis of the gaps should be provided.

Response：I will expand the introduction and literature review to include other game types such as Nash games, differential games, and fuzzy games. I will also provide an analysis of the gaps in the existing literature.

4.) The primary content of the manuscript ought to advocate for the following: methodology, theoretical outcomes, and game model construction need to be explicitly and carefully described. The present form appears to be unacceptable.

Response：Thank you for your feedback. I will reorganize the methodology section to present the theoretical outcomes and game model construction more explicitly and carefully. I will ensure that the logic and structure are clear and detailed.

5.) The paper lacks any numerical findings. To ensure that the proposed pricing model is important to real-world scenarios, a case study is suggested here.

Response：The paper describes a scenario for rail freight option trading in China. Due to the relatively closed nature of the rail freight option market in China, conducting relevant experiments has been challenging. However, I will include a case study to demonstrate the applicability of the proposed pricing model to real-world scenarios.

6.) A discourse section addressing the model's contributions, limitations, and challenges along with potential solutions is absent. A more comprehensive analysis of the managerial implications and potential avenues for future research was also expected.

Response:I will add a discussion section addressing the model's contributions, limitations, and challenges, along with potential solutions. I will also provide a more comprehensive analysis of the managerial implications and suggest avenues for future research.

Reviewer #2: The data used in the manuscript is rigorous and reliable, which is sufficient to support the existing research conclusions. Moreover, appropriate statistical methods are adopted. From data to methods, they can support the conclusion of the manuscript. In addition, the writing of the manuscript adopts relatively standard English expression

Response：Thanks for your evaluation and i will rectify my manuscript as your suggestion!

Reviewer #3: The manuscript presents a generally well-structured argument and the overall flow of ideas is commendable. However, there are a few areas that require attention:

Logical Coherence in Sentences: There are instances where the logic within individual sentences appears to be flawed. It would be beneficial to revise these sentences for clarity and precision.

Response：thanks for your suggestion and i will reconstruct my logical coherence in sentences

Consistency in Sentence Structure: The manuscript exhibits a mix of long and short sentences, which can disrupt the reading flow. A more balanced approach to sentence construction is recommended to ensure consistency.

Response: Thank you for your suggestion. I will ensure consistency in sentence structure as you advised.

Content Distribution: Some sections seem to have an uneven distribution of content, with certain areas lacking the depth required to fully support the arguments being made.

Response:Thank you for your suggestion. I will balance the content distribution as you recommended.

Connectivity Between Formulas: The transitions between mathematical formulas are not as seamless as they could be. It is important to ensure that the relationships between formulas are clearly articulated to maintain the integrity of the technical narrative.

To enhance the manuscript's quality, I suggest a thorough review of the identified areas, focusing on refining sentence logic, maintaining structural consistency, balancing content distribution, and improving the connectivity of mathematical expressions.

Response:Thank you for your suggestion. I will improve the connectivity between formulas as you advised.
