# Supplementary material for: A Stackelberg-based repurchase strategy for rail freight options (BRFO)
Source: PLoS One. 2024 Sep 20;19(9):e0307215. doi: 10.1371/journal.pone.0307215 (PMC11414986; doi:10.1371/journal.pone.0307215)
Supplement: Attachment — Submitted filename: Response.docx [file pone.0307215.s004.docx]

Reviewer #1: The authors partially considered my comments and missed some of them:
1.) Provide importance to your work, including its challenges and advantages as well as drawbacks.

Response: We appreciate the reviewer’s suggestion to highlight the importance of our work more clearly. In the revised manuscript, we have added a section in the introduction that outlines the significance of the BRFO model. This includes a detailed discussion of the challenges it addresses, such as information asymmetry and market uncertainty, as well as its advantages, including dynamic pricing and strategic benefits for railway enterprises. We have also acknowledged potential drawbacks and limitations to provide a balanced view.

2.) Present your work clearly in Section 3; the current version is not acceptable! Present your methodology with a flowchart.

Response: We apologize for any lack of clarity in Section 3. In the revised manuscript, we have restructured Section 3 for better clarity and readability. We have also included a flowchart that visually represents our methodology. This flowchart outlines the steps involved in integrating the multi-phase trigeminal tree pricing model and the non-parametric Itô stochastic method, providing a clearer understanding of our approach.

3.) Section 4 should be revised; write each equation in a clear and rigorous manner.

Response: Thank you for pointing this out. We have revised Section 4 to ensure that each equation is presented in a clear and rigorous manner. This includes providing detailed explanations for each step in the derivation of the equations and ensuring that all notations are consistently used throughout the section. These revisions aim to enhance the comprehensibility and accuracy of our mathematical presentation.

4.) Include a numerical example; a real-world scenario is recommended for your model to test the robustness and effectiveness of your work.

Response: We have added a numerical example in the revised manuscript to illustrate the application of the BRFO model. This example is based on a real-world scenario in the rail freight industry, demonstrating how our model can be used to manage rail freight transactions. The example includes data and results that showcase the robustness and effectiveness of our proposed methodology.
5.) Provide a sensitivity analysis of the parameters of your model in the numerical section.

Response: We have included a sensitivity analysis in the numerical section of the revised manuscript. This analysis examines the impact of key parameters on the model’s performance, providing insights into the stability and reliability of our results under different conditions. The sensitivity analysis helps to identify critical parameters and their influence on the outcomes of the BRFO model.
6.) Include a discussion section for your theoretical and numerical conclusions as well as future research topics.

Response: We have added a discussion section in the revised manuscript. This section synthesizes our theoretical and numerical findings, highlighting the implications of our results for the strategic management of rail freight transactions. Additionally, we have outlined potential areas for future research, suggesting ways to extend and refine our model to address emerging challenges in the field.

Reviewer #2: Summary:

I have carefully reviewed the revised manuscript titled "A Stackelberg-Based Repurchase Strategy for Rail Freight Options (BRFO)" and am pleased to report that the authors have effectively addressed all the concerns raised in my initial review. The revisions have significantly enhanced the clarity, methodological rigor, and overall contribution of the study.

Detailed Comments:

Addressing Previous Concerns: The authors have successfully resolved the issues highlighted in the initial review. The integration of traditional buyback theory with a multi-phase trigeminal tree pricing model and parameter identification through a nonparametric Ito stochastic method is now more clearly explained, addressing the concerns of methodological transparency and robustness.

Methodological Clarity: The authors have provided a detailed explanation of their methodology, including the steps taken to mitigate information asymmetry and market uncertainty. This added clarity greatly enhances the reader’s understanding and confidence in the study’s outcomes.

Data and Analysis: The inclusion of additional data and more comprehensive statistical analyses strengthens the manuscript. The dynamic pricing strategies and market adaptation mechanisms are well-supported by the data, making the findings more compelling.

Literature Review and Contextualization: The revised manuscript includes a more thorough literature review, effectively contextualizing the study within the broader field of railway freight pricing and strategic management. This helps to highlight the novelty and significance of the proposed Buyback Rail Freight Option (BRFO).

Presentation and Organization: The overall presentation of the manuscript has improved. The figures and tables are more informative and easier to interpret, and the organization of the manuscript allows for a more logical flow of information. The construction of a Nash equilibrium framework within the railway freight pricing context is particularly well-presented.

Conclusion:

Given the substantial improvements and the thorough manner in which the authors have addressed the concerns raised, I recommend that the manuscript be accepted for publication. The study presents a valuable contribution to the field of rail freight transactions and strategic management, and will be of significant interest to the readership.

Response: Thank you for acknowledging our efforts to improve the methodological transparency and robustness. We have worked diligently to ensure that the integration of traditional buyback theory with our pricing model and stochastic method is clearly explained.

Reviewer #3: 1.Please clearly define the contributions of your paper.

Response: Thank you for your valuable feedback. In the revised manuscript, we have added a dedicated section in the introduction to clearly outline the key contributions of our paper. Specifically, we emphasize:

The introduction of a novel Buyback Rail Freight Option (BRFO) that leverages Stackelberg game theory to address strategic management challenges in rail freight transactions.

The integration of traditional buyback theory with a multi-phase trigeminal tree pricing model and a non-parametric Itô stochastic method to tackle issues of information asymmetry and market uncertainty.

The development of dynamic pricing strategies and market adaptation mechanisms that provide significant strategic benefits for railway enterprises.

The construction of a Nash equilibrium framework within the railway freight pricing context.

We believe these additions will provide a clearer understanding of the unique contributions our study makes to the field.

2. There are some grammatical errors in this paper; please review and revise the entire text carefully.

Response: We appreciate your attention to the language quality of our manuscript. We have thoroughly reviewed the entire text and have made necessary revisions to correct grammatical errors and improve overall readability.

2.There are some grammatical errors in this paper; please review and revise the entire text carefully.

Response: Thank you for pointing out the grammatical errors. We have conducted a thorough review of the entire manuscript to identify and correct these errors.

Reviewer #4: Given the manuscript's notable strengths and the authors' thorough approach in addressing the research problem, I recommend it for publication. The study presents an innovative Buyback Rail Freight Option (BRFO), leveraging Stackelberg game theory to enhance strategic management in rail freight transactions. By integrating traditional buyback theory with a multi-phase trigeminal tree pricing model and a nonparametric Ito stochastic method, the authors effectively address information asymmetry and market uncertainty. The resulting dynamic pricing strategies and market adaptation mechanisms offer significant strategic benefits for railway enterprises. This research is a valuable addition to the field and will be of great interest to the journal’s readership.

Response: We are grateful for your positive feedback and recommendation for publication. We appreciate your recognition of the strengths of our manuscript and the thorough approach we have taken in addressing the research problem.
